# The Effect of a Caffeine and Nicotine Combination on Nicotine Withdrawal Syndrome in Mice

**DOI:** 10.3390/nu16183048

**Published:** 2024-09-10

**Authors:** Zhe Chen, Naiyan Lu, Xu Li, Qingrun Liu, Yujie Li, Xiyue Li, Ximiao Yu, Haotian Zhao, Chang Liu, Xue Tang, Xun Wang, Weisun Huang

**Affiliations:** 1 Department of Oncology, The Affiliated Wuxi People’s Hospital of Nanjing Medical University, Wuxi People’s Hospital, Wuxi Medical Center, Nanjing Medical University, Wuxi 214023, China; 7231207002@stu.jiangnan.edu.cn; 2 School of Food Science and Technology, Jiangnan University, Wuxi 214122, China; lunaiyan@jiangnan.edu.cn (N.L.); lixu97@126.com (X.L.); 7210112094@stu.jiangnan.edu.cn (Q.L.); tangxue@jiangnan.edu.cn (X.T.); 3 Sports and Health Research Institute, Jiangnan University, Wuxi 214122, China; haotianzhao@jiangnan.edu.cn; 4 School of Chemical Engineering and Technology, Tianjin University, Tianjin 300072, China; liyujiehf@tju.edu.cn; 5 Department of Pulmonary and Critical Care Medicine, Jiangnan University Medical Center, Jiangnan University, Wuxi 214126, China; 17851302010@163.com (X.L.); 6232825034@stu.jiangnan.edu.cn (X.Y.); 081104125@fudan.edu.cn (X.W.); 6 School of Sport Science, Beijing Sport University, Beijing 100084, China; c.liu@bsu.edu.cn

**Keywords:** caffeine, nicotine, withdrawal effect, behavior, dopamine

## Abstract

Nicotine dependence is an important cause of excessive exposure to tobacco combustion compounds in most smokers. Nicotine replacement therapy is the main method to treat nicotine dependence, but it still has its shortcomings, such as the inability to mitigate withdrawal effects and limited applicability. It has been hypothesized that a combination of low-dose nicotine and caffeine could achieve the same psychological stimulation effect as a high dose of nicotine without causing nicotine withdrawal effects. To establish a model of nicotine dependence, male C57BL/6J mice were subcutaneously injected four times a day with nicotine (2 mg/kg) for 15 days and fed with water containing nicotine at the same time. They were randomly divided into four groups. After 24 h of withdrawal, different groups were injected with saline, nicotine (0.25 mg/kg or 0.1 mg/kg), or nicotine (0.1 mg/kg) and caffeine (20 mg/kg). Behavioral and physiological changes were evaluated by an assessment of physical signs, open field tests, elevated plus maze experiments, forced swimming tests, hot plate tests, and new-object-recognition tests. The changes in dopamine release in the prefrontal cortex (PFC) and ventral tegmental area (VTA) in the midbrain were analyzed using ELISA. The results showed that a combination of caffeine and nicotine could effectively relieve nicotine withdrawal syndrome, increase movement ability and pain thresholds, reduce anxiety and depression, enhance memory and cognitive ability, and increase the level of dopamine release in the PFC and VTA. Thus, caffeine combined with nicotine has potential as a stable and effective treatment option to help humans with smoking cessation.

## 1. Introduction

Nicotine dependence makes most smokers overexposed to tobacco-burning compounds, which leads to preventable diseases and premature death [1,2]. A major challenge in fighting nicotine dependence is withdrawal syndrome, which has various unendurable physical and psychological symptoms that lead to a high rate of relapse [3]. The most common intervention method for nicotine dependence is nicotine replacement therapy. This method can effectively reduce the blood concentration of nicotine, but it is not suitable for those who have cardiovascular disease, moderate fatty liver, or gastric ulcers. Because nicotine replacement therapy can lead to considerable withdrawal effects [4,5], there is a need for new methods to address nicotine dependence.

Nicotine has short-term cognitive enhancement effects. Its capacity to cross the blood–brain barrier, activating neuronal nicotinic acetylcholine receptors (nAChRs) in the mesolimbic dopamine system, enhances neurotransmission in the ventral tegmental area (VTA) and nucleus accumbens [6,7,8]. In addition, nicotine stimulates nAChRs in glutamatergic and GABA terminals. This stimulation leads to the release of glutamate, an excitatory neurotransmitter, and also increases the levels of GABA, which is an inhibitory neurotransmitter. Compared to those on the glutamate neuron, nAChRs desensitize at a faster rate on the GABA neuron. These reward mechanisms of the brain undergo neural adaptation after long-term exposure to nicotine in tobacco products, which is the basis for nicotine dependence [9]. However, chronic nicotine exposure results in numerous neuroadaptations, such as the upregulation of specific nAChR subtypes, and withdrawal syndrome occurs when nicotine is no longer present [10,11,12]. The symptoms of nicotine withdrawal in humans include anxiety, depression, irritability, increased appetite, insomnia, and restlessness. In mice, withdrawal symptoms can be evaluated based on physical symptoms (increased tremors, shaking, scratching, and sensitivity to pain) and emotional symptoms (anxiety and depression), which can be explored through various behavioral analyses. Nicotine withdrawal symptoms in mice typically peak between 12 and 36 h after withdrawal [10]. Chronic nicotine exposure decreases baseline dopamine levels in the nucleus accumbens and prefrontal cortex (PFC), potentially leading to strong withdrawal effects.

Caffeine results in mild mental stimulation and has similar effects of improving attention and decreasing fatigue to those of nicotine [13,14]. However, unlike nicotine, caffeine’s psychostimulant effects result from blocking A2A receptors, which allows dopamine to activate D2 receptors in the mesolimbic system more effectively [15,16]. Because caffeine does not lead to acute modulation of dopamine release, it rarely results in strong dependence and withdrawal effects as in the case of nicotine, and it can be safely consumed through tea, coffee, and soft drinks [17,18].

Therefore, a combination of a low dosage of nicotine and caffeine could potentially achieve the same psychostimulant effect as a high dosage of nicotine without triggering nicotine withdrawal effects. To test this hypothesis, behavioral and physiological studies were carried out to investigate the effects of such combinations in mice with nicotine withdrawal. The results could help to find a more tolerable yet effective way to help people to quit smoking.

## 2. Materials and Methods

### 2.1. Animals

Experiments were performed with 48 male specific-pathogen-free C57BL/6J mice aged 8–9 weeks (initial weight: 21–22 g) (Zhejiang Viton Lihua Laboratory Animal Technology Co., Ltd., Jiaxing, China). Their enclosure conditions included a controlled ambient temperature of 25 ± 2 °C, relative humidity of 50–70%, and 12 h of alternating light and dark light conditions. The animal experiments were carried out with the approval of the Ethics Committee of the Animal Experimentation Centre of Jiangnan University (approval number: JN. No20220615c0320825). All animal experiment protocols followed the guidelines of the National Institutes of Health (NIH).

### 2.2. Experimental Reagents and Equipment

Experiments were conducted using nicotine tartrate (purity ≥ 98%) (Shanghai Aladdin Biochemical Technology Co., Ltd., Shanghai, China), caffeine (purity ≥ 98%) (Purely Excellent Biotechnology Co., Ltd., Shanghai, China), and a mouse dopamine ELISA kit (Huijia Biotechnology Co., Ltd., Xiamen, China). EthoVision XT 11.5 software was used for analysis (Noldus Information Technology, Wageningen, The Netherlands), and a DS-2CD2TDY-1 4 mm network camera was used to observe the mice (Hikvision Digital Technology Co., Ltd., Hangzhou, China). The rest of the behavioral equipment was provided by the Jiangnan University Animal Experiment Centre.

### 2.3. Experimental Design

The experimental design was informed by the pertinent literature [19,20,21,22,23]. The mice were randomly divided into 4 groups. The MOD group received a subcutaneous injection of physiological saline, while the H-NIC group received a nicotine solution at a dose of 0.25 mg/kg, the L-NIC group received the nicotine solution at a dose of 0.1 mg/kg, and the L-NIC+CAF group received nicotine (0.1 mg/kg) and caffeine (20 mg/kg). There were 12 mice in each group, of which 6 were used for behavioral experiments, while the other 6 were used for biochemical analysis. All mice were assessed for physical signs and scored prior to modeling nicotine dependence. 

To establish the nicotine dependence model, a nicotine solution was made by dissolving nicotine tartrate in saline; it was injected into the mice subcutaneously for 15 consecutive days. The injections were performed four times a day (8:00–20:00 at 4 h intervals) at a dose of 2 mg/kg (measured as free nicotine base). Furthermore, nicotine tartrate (200 μg/mL) and saccharin sodium (3 mg/mL) were added to the drinking water [24].

Withdrawal was established for 24 h after giving the last dose of nicotine to the mice. The mice were transferred to the behavioral laboratory at 2 h before experiments, and then the experimental treatments were administered 10 min before behavioral experiments. For biochemical analysis, after 10 min of drug administration, the PFC and the ventral tegmental area (VTA) of the brain were removed, placed on ice, snap-frozen in liquid nitrogen, and transferred to a refrigerator kept at −80 °C for storage.

### 2.4. Assessment of Physical Signs

Physical signs were assessed in nicotine-dependent mice according to the method of Castañé et al. [25]. For 5 min, the number of incidences of wet-dog shakes, forepaw tremor, sniffing, standing, and scratching was recorded as x. The number of incidences of drooping eyelids, genital licking, tremor, penile erection, and teeth chattering was recorded as y. Finally, the overall physical sign score was calculated as 0.5 × x + 1 × y. A higher score indicated that the mice had a more obvious withdrawal response.

### 2.5. Open Field Test (OFT)

An OFT was conducted according to the method of Ueno et al. [26] with slight modifications. The open field was a 40 cm × 40 cm × 35 cm box with an upper opening that was divided at the bottom into a central area (20 cm × 20 cm) and a marginal area. The movements of the mice were continuously recorded for 10 min, and the mice’s movement distance, rest time, times entering the central area, and times dwelling in the central and marginal areas were analyzed.

### 2.6. Elevated Plus Maze (EPM) Experiment

The EPM device was composed of two perpendicular open arms and a closed arm of 30 cm × 6 cm at 50 cm above the ground. There was also a 6 cm × 6 cm platform in the center. The mice’s movements were observed for 5 min, and the proportion of times entering the open arm (PEO) and the proportion of time dwelling in the open arm (PDO) were calculated. More serious anxiety was indicated by a decrease in the time of dwelling in the open arm.

### 2.7. Forced Swimming Test (FST)

Referring to the method of Can et al. [27], a cylindrical bucket with a height of 30 cm and an inner diameter of 11 cm was filled with water to a level of 20 cm to ensure that the hind limbs of the mice could not be supported by or touch the bottom of the bucket. The temperature of the water was maintained at 25 ± 1 °C. The mice were placed into the bucket and observed to swim for 10 min, and then their immobilization time in the last 3 min was analyzed using EthoVision XT 11.5. More depression was indicated by a long immobilization time.

### 2.8. Hot Plate Test (HPT)

Referring to the method of Alijanpour et al. [28], with slight modifications, the temperature of a hot plate was maintained at 52 °C, and a transparent acrylic cylinder was placed on the hot plate to keep the mice on the plate. The mice were timed immediately after contacting the hot plate. Acute responses such as jumping and licking the hindfoot were considered as indicators of pain perception, and the timing was stopped to obtain the pain latency.

### 2.9. New Object Recognition Experiment (NOR)

According to the method of Chang et al. [29], with slight modifications, an experiment to examine new object recognition was conducted in 3 stages. In the first stage, the mice were placed in a box without objects, allowed to explore freely for 5 min, and then removed to rest for 20 min. These operations were repeated 3 times. In the second stage, two identical objects (objects A1 and A2) were placed in the box. The mice were placed in the box and allowed to explore for 10 min. In the third stage, A2 was replaced by a different object (object B), and then the mice were placed in the box. A camera was used to record the time that the mice spent on the exploration of the two objects for 10 min. Exploration of the object was considered as touching of nose tips to the object and approaching the object within about 2 cm.

### 2.10. Determination of Dopamine Content

Pre-cooled phosphate-buffered saline was used as a medium to prepare a homogenate of brain tissue at a 1:10 (m:v) ratio. The mixture was centrifuged, and then the supernatant was collected. The dopamine content was determined according to the instructions of the mouse dopamine ELISA kit and quantified by protein concentration.

### 2.11. Statistical and Analysis

SPSS 20.0 was used for statistical analysis. The statistical significance of the results was confirmed at different confidence levels (* *p* < 0.05, ** *p* < 0.01, *** *p* < 0.001, and **** *p* < 0.0001; ns indicates no significant difference). The Shapiro–Wilk test was used to analyze the normal distribution. If the data conformed to the normal distribution and had similar variance, one-way ANOVA was used; if the data conformed to the normal distribution but had different variances, the Brown–Forsythe ANOVA test was used. GraphPad Prism (Version 9.0, Boston, MA, USA) was used to plot data. 

## 3. Results

### 3.1. Physical Signs and Behavior in OFT

**Figure 1:**
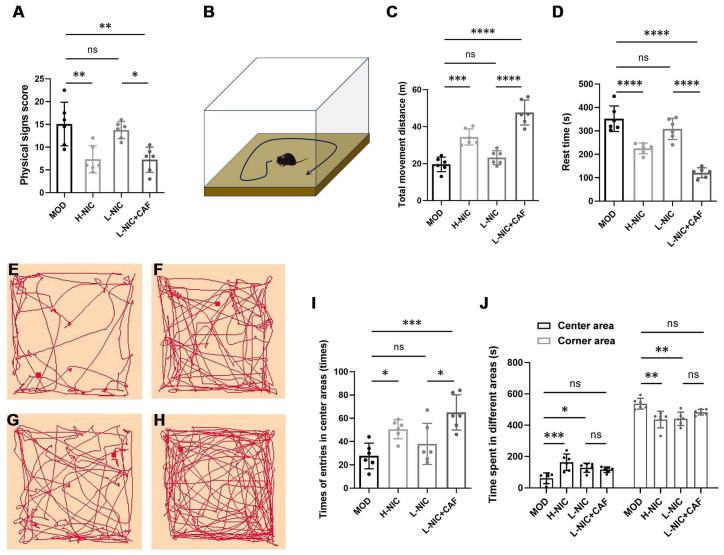
Effect of caffeine combined with nicotine on the physical signs and behavior of mice with nicotine withdrawal. (**A**) Physical sign score (F_(3, 20)_ = 9.665, *p* < 0.001). (**B**) Diagram of OFT. (**C**) Total movement distance (F_(3, 20)_ = 40.70, *p* < 0.0001). (**D**) Rest time (F_(3, 20)_ = 41.17, *p* < 0.0001). Movement track of mice in the last 5 min of OFT for different groups: (**E**) MOD, (**F**) H-NIC, (**G**) L-NIC, and (**H**) L-NIC+CAF. (**I**) Number of entries in center areas (F_(3, 20)_ = 8.539, *p* < 0.001). (**J**) Time spent in center and corner areas (F_(3, 20)_ = 8.615, *p* < 0.001; F_(3, 20)_ = 8.735, *p* < 0.001). MOD: Mice that received physiological saline. H-NIC: Mice that received nicotine solution at 0.25 mg/kg. L-NIC: Mice that received nicotine solution at 0.1 mg/kg. L-NIC+CAF: Mice that received mixture of nicotine and caffeine at 0.1 and 20 mg/kg, respectively. * *p* < 0.05, ** *p* < 0.01, *** *p* < 0.001, **** *p* < 0.0001; ns: no significant difference.

The physical sign scores of mice are shown in Figure 1A. The physical sign score of the MOD group was 15.08 ± 4.78. After subcutaneous injection of high-dose nicotine, the physical sign score of the H-NIC group decreased to 7.33 ± 2.96, and that of the L-NIC group injected with low-dose nicotine decreased to 13.75 ± 1.89. The score of the L-NIC-CAF group decreased to 7.25 ± 2.75, which was the lowest among all groups.

The movements of each group of mice in the open field are shown in Figure 1B–H. In the MOD group, the total distance of horizontal movement was the shortest, and the resting time was the longest (19.60 ± 3.93 m and 351.98 ± 49.89 s, respectively). After the high-dose injection, the H-NIC group showed a significant difference from the MOD group (*p* < 0.001), with a total horizontal movement distance of 34.43 ± 4.37 m and a rest time of 225.26 ± 20.97 s. The total distance of horizontal movement of the L-NIC group was slightly higher than that of the MOD group (23.26 ± 3.72 m), and its rest time was slightly shorter (308.33 ± 41.38 s). The L-NIC+CAF group had the longest total horizontal movement distance (47.65 ± 6.76 m) and the shortest rest time (121.24 ± 19.41 s). The movement distance was 2.05 times that of the L-NIC group, while the rest time was 39.32% shorter.

Figure 1I,J show the time spent in the center and margins of the open field and the number of times that mice entered the central area in each group. The mice in the MOD group entered the central area the least frequently (27.67 ± 10.98 times). After subcutaneous injection of nicotine, the number of times that the mice entered the central region increased to 50.50 ± 8.22 times in the H-NIC group and to 38.00 ± 17.66 times in the L-NIC group. The L-NIC+CAF group entered the central region the most frequently at 65.00 ± 15.16 times. This number is 1.71 times that of the L-NIC group. The MOD group had the shortest time of dwelling in the central area (61.75 ± 34.34 s) and the longest time of dwelling in the corner area (537.17 ± 34.38 s). All other groups spent a longer amount of time in the central region and shorter amount of time in the marginal region.

### 3.2. Anxiety Behavior in EPM and Depressive Behavior during FST

**Figure 2:**
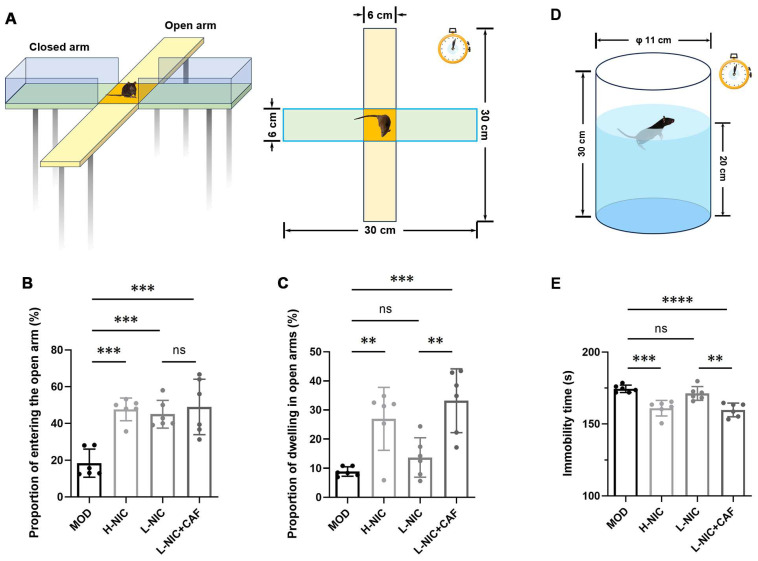
Effect of caffeine combined with nicotine on anxiety behavior and depressive behavior of mice with nicotine withdrawal. (**A**) Diagram of EPM (left: front view, right: top view). (**B**) Proportion of times they entered the open arms (PEO, F_(3, 20)_ = 13.12, *p* < 0.0001). (**C**) Proportion of time they dwelled in open arms (PDO, F_(3, 20)_ = 10.76, *p* < 0.001). (**D**) Diagram of FST. (**E**) Immobility time in FST (F_(3, 20)_ = 15.80, *p* < 0.0001). MOD: Mice that received physiological saline. H-NIC: Mice that received nicotine solution at 0.25 mg/kg. L-NIC: Mice that received nicotine solution at 0.1 mg/kg. L-NIC+CAF: Mice that received mixture of nicotine and caffeine at 0.1 and 20 mg/kg, respectively. ** *p* < 0.01, *** *p* < 0.001, **** *p* < 0.0001; ns: no significant difference.

The EPM is illustrated in Figure 2A. Figure 2B,C show that the PEO and PDO of the MOD group were 18.45 ± 7.66% and 8.88 ± 1.62%, respectively, which were the lowest among all groups. The PEO and PDO of the H-NIC group increased significantly (47.63 ± 6.20% and 26.99 ± 10.80%). There was also an increase in the PEO of the L-NIC group to 45.01 ± 7.55%, while the PDO increased to 13.68 ± 6.75%. The L-NIC-CAF group had the highest PEO and PDO among all groups at 49.00 ± 15.14% and 33.17 ± 10.99%, respectively. The PDO of the L-NIC-CAF group was 2.42 times that of the L-NIC group.

Figure 2D,E show the immobility time of each group of mice after being forced to swim. The immobility time of mice in the MOD group was the longest (174.47 ± 2.57 s). After different doses of nicotine were given, the immobility time of the H-NIC group and the L-NIC group decreased to 160.93 ± 5.43 s and 171.26 ± 4.82 s, respectively. The immobility time of the L-NIC+CAF group (159.74 ± 4.81 s) was the lowest among all groups and was 0.93 times that of the L-NIC group.

### 3.3. Pain Latency in HPT

**Figure 3:**
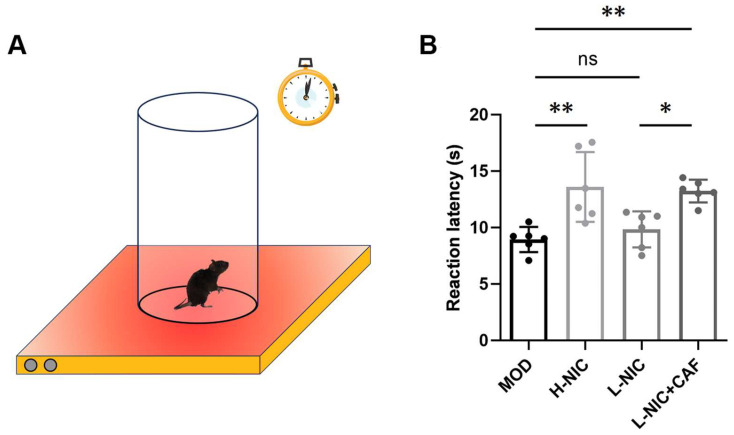
Effect of caffeine combined with nicotine on pain latency of mice with nicotine withdrawal. (**A**) Diagram of HPT. (**B**) Pain latency in HPT (F_(3, 20)_ = 9.306, *p* < 0.001). MOD: Mice that received physiological saline. H-NIC: Mice that received nicotine solution at 0.25 mg/kg. L-NIC: Mice that received nicotine solution at 0.1 mg/kg. L-NIC+CAF: Mice that received mixture of nicotine and caffeine at 0.1 and 20 mg/kg, respectively. * *p* < 0.05, ** *p* < 0.01; ns: no significant difference.

The results of HPT in each group of mice are shown in Figure 3A,B. The pain latency was 8.94 ± 1.12 s in the MOD group and was higher at 13.60 ± 3.09 s in the H-NIC group. The pain latency of the L-NIC group was slightly higher at 9.84 ± 1.60 s compared to the MOD group. The pain latency of the L-NIC-CAF group was 13.23 ± 1.00 s, which was significantly higher than that of the L-NIC group (*p* < 0.05). The pain latency of the L-NIC-CAF group was 1.34 times that of the L-NIC group.

### 3.4. Cognitive Behavior in NOR

**Figure 4:**
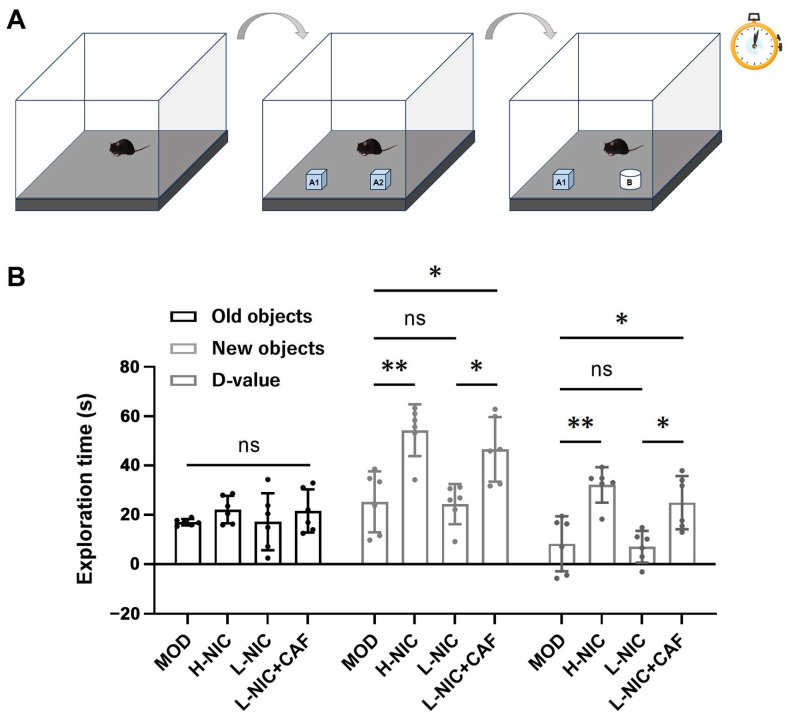
Effect of caffeine combined with nicotine on cognitive behavior of mice with nicotine withdrawal. (**A**) Diagram of NOR. (**B**) Exploration time of mice in NOR (D-value: difference value. F_(3, 11.93)_ = 0.7628, *p* = 0.5365; F_(3, 20)_ = 10.96, *p* < 0.001; F_(3, 20)_ = 11.05, *p* < 0.001). MOD: Mice that received physiological saline. H-NIC: Mice that received nicotine solution at 0.25 mg/kg. L-NIC: Mice that received nicotine solution at 0.1 mg/kg. L-NIC+CAF: Mice that received mixture of nicotine and caffeine at 0.1 and 20 mg/kg, respectively. * *p* < 0.05, ** *p* < 0.01; ns: no significant difference.

Each group’s exploration time of new and old objects are shown in Figure 4A,B. The exploration time of new and familiar objects of mice with nicotine withdrawal were 17.00 ± 1.32 s and 25.32 ± 12.33 s with a difference value of 8.31 ± 11.13 s. Compared with the MOD group, the exploration time of the H-NIC mice increased to 54.27 ± 10.48 s, and the difference value was 32.11 ± 7.17 s. The difference in time for exploring objects between the L-NIC group and the MOD group was relatively small. The difference value of the L-NIC group was 7.15 ± 6.40 s. The exploration time of the L-NIC+CAF group for new objects was increased to 46.56 ± 13.10 s. The difference value of this group was 24.98 ± 10.77 s, which was 3.49 times that of the L-NIC group.

### 3.5. Dopamine Levels

**Figure 5:**
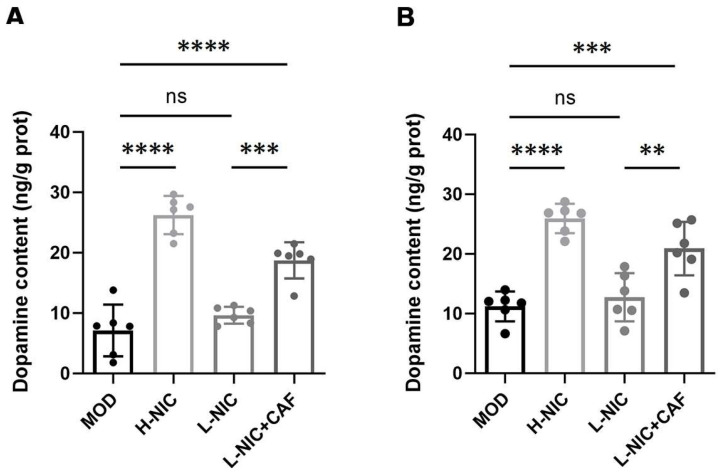
Effect of caffeine combined with nicotine on dopamine levels in the brain of withdrawal mice. (**A**) PFC (F_(3, 20)_ = 46.78, *p* < 0.0001). (**B**) VTA (F_(3, 20)_ = 23.82, *p* < 0.0001). MOD: Mice that received physiological saline. H-NIC: Mice that received nicotine solution at 0.25 mg/kg. L-NIC: Mice that received nicotine solution at 0.1 mg/kg. L-NIC+CAF: Mice that received mixture of nicotine and caffeine at 0.1 and 20 mg/kg, respectively. ** *p* < 0.01, *** *p* < 0.001, **** *p* < 0.0001; ns: no significant difference.

Figure 5 shows the effects of caffeine combined with nicotine on dopamine levels in different brain regions. Acute nicotine administration increased dopamine levels in the PFC in a dose-dependent manner (Figure 5A). The dopamine level was 7.13 ± 4.27 ng/g protein in the MOD group and increased to 26.25 ± 3.17 ng/g protein in the H-NIC group. The dopamine level of the L-NIC group increased to 9.66 ± 1.40 ng/g protein. The dopamine level in the PFC of the L-NIC+CAF group was 18.75 ± 3.01 ng/g protein, which is 1.94 times that of the L-NIC group.

The dopamine levels in the VTA also showed a similar trend (Figure 5B). Compared with the MOD group’s dopamine level (11.22 ± 2.49 ng/g protein), the level of the H-NIC group increased to 25.95 ± 2.46 ng/g protein. The level of the L-NIC group increased to 12.74 ± 4.03 ng/g protein. The level in the L-NIC+CAF group was 20.91 ± 4.49 ng/g protein, which is 1.64 times that of the L-NIC group (*p* < 0.01).

## 4. Discussion

Long-term intake of nicotine can lead to many neuroadaptations, and withdrawal syndrome occurs when nicotine is no longer present. Caffeine is one of the main active ingredients in coffee, tea, and soft drinks and causes mild mental stimulation. Therefore, partially replacing nicotine with caffeine may help alleviate withdrawal syndrome caused by limiting nicotine intake, and behavioral changes in mice were monitored to test this.

The physical sign score of the MOD group was higher than that of the other groups, suggesting that nicotine deficiency leads to severe withdrawal syndrome in nicotine-addicted mice. There was no significant difference between the L-NIC group and MOD group, indicating that low nicotine intake had a limited effect on the relief of withdrawal syndrome. The score in the L-NIC+CAF group was the lowest among the groups. Compared with the L-NIC group, the addition of caffeine in the L-NIC+CAF group with the same low dose of nicotine showed significant alleviation of the physical signs caused by withdrawal syndrome (*p* < 0.05). Some studies suggest that caffeine increases dopamine release and the number of dopamine receptors in the striatum through A2A receptor antagonism. This may be the reason why caffeine can alleviate the adverse reactions of nicotine withdrawal [30,31].

In the OFT, the total distance of horizontal movement in the MOD group was the shortest, and the resting time was the longest. This indicated that the movement ability of nicotine-addicted mice was lower without nicotine, which is consistent with a study by Rud et al. [32]. When nicotine was subcutaneously injected, the mice showed an increase in horizontal movement distance and a decrease in rest time in a dose-dependent manner. Improvement in the low-dose injection group was limited. Caffeine combined with nicotine significantly improved the low movement ability of mice with nicotine withdrawal. The improvement was significant compared with the result of the L-NIC group (*p* < 0.0001) and was also better than that of the H-NIC group.

Mice are generally considered more anxious if they prefer to stay in the marginal region rather than the central region in an OFT [33]. Figure 1I,J show that the combination of caffeine and nicotine increased the number of times that the mice entered the central area, which was the highest among all the groups and significantly higher than that of the L-NIC group (*p* < 0.05). These results suggested that the combination of caffeine and nicotine significantly improved anxiety. In addition, the mice’s dwelling time was longer in the central area and shorter in the marginal area, which indicated a stronger desire to explore and more willingness to move around in the box, as shown in Figure 1C–H.

The EPM experiment has value for evaluating “emotion” in mice, especially in terms of anxiety-related behaviors [34]. The test is based on rodents’ natural tendency to explore novel environments and their natural avoidance of unprotected, bright, and high places (represented by open arms) [35,36]. In this study, the PEO and PDO were used to evaluate anxiety levels (Figure 2A–C). More anxious mice have lower PEO and PDO scores. The mice in the MOD group showed the lowest PEO and PDO, indicating anxiety, which is consistent with the results of Bagosi et al. [37].

The PEO was increased in the L-NIC group, but there was no significant difference in the PDO between the L-NIC group and the MOD group. This may be due to insufficient nicotine intake; therefore, the mice still showed symptoms of anxiety. Although they had more willingness to explore open arms, they did not stay there very long. Importantly, PEO and PDO in the L-NIC+CAF group were the highest among all groups. The L-NIC+CAF group showed a significant increase in the PDO compared to the L-NIC group (*p* < 0.01), indicating that the combination of caffeine and nicotine could effectively alleviate anxiety in mice with nicotine withdrawal and increase their curiosity.

The FST is one of the most commonly used behavioral methods to evaluate depressive behavior in animals [38]. The MOD group had the longest immobility time and, thus, the highest depression tendency. This is consistent with the results of Bagosi et al. [37] and Mannucci et al. [39], who showed that nicotine-addicted mice had a longer immobility time after abstinence due to the depression caused by the realization that they could not escape. After different doses of nicotine were given to mice with nicotine withdrawal, the depressive behaviors of the H-NIC group and the L-NIC group were alleviated. The L-NIC-CAF group had the lowest immobility time, which was 0.93 times that of the L-NIC group (*p* < 0.01). These results indicated that caffeine combined with nicotine could significantly alleviate depression in mice during nicotine withdrawal.

Some studies have shown that smokers become more sensitive to pain when quitting smoking and that pain sensitivity is positively correlated with the degree of nicotine withdrawal syndrome [40,41]. Therefore, the response of mice to pain was tested by an HPT. The results showed that the pain latency of the L-NIC+CAF group was significantly higher than that of the MOD group (*p* < 0.01) and the L-NIC group (*p* < 0.05). This is consistent with the findings of Nastase et al. [42], who indicated that caffeine is an analgesic adjuvant strengthening the analgesic effect of nicotine. Caffeine alleviates pain by blocking adenosine receptors, which affect pain signals, or by blocking peripheral adenosine receptors, which affect sensory afferents [43,44]. These results suggested that caffeine combined with nicotine could increase the pain threshold of mice with nicotine withdrawal and reduce pain.

The memory ability of the mice was tested through new object recognition. Memory ability can be measured by the difference between the exploration time of a new object and a familiar object [45]. A larger difference indicates that animals prefer to spend more time exploring a new object rather than a familiar one and that they have a stronger memory ability and preference for novel things.

Figure 4 shows that the exploration time for the new object in all groups was longer than that of the familiar object, and there was no significant difference in the exploration time of the old object. Interestingly, there was no significant difference in the time of exploring the new object between the L-NIC group and the MOD group. However, the time of exploring the new object in the L-NIC+CAF group was significantly increased to 46.56 ± 13.10 s (*p* < 0.05), and the d-value was 3.49 times that of the L-NIC group. These results suggested that the combination of caffeine and nicotine could enhance the memory ability and exploration desire of mice with nicotine withdrawal, as well as their time spent exploring a new object.

Finally, we investigated the effect on dopamine levels (Figure 5). As a neuromodulator, dopamine affects the function of the PFC by enhancing or inhibiting synaptic information transmission [46]. The PFC participates in working memory, attention, emotional control, and action decision [47] and receives dopamine input from other brain regions, including the VTA. The circuit from the VTA to the PFC plays an important role in dependence, depression, and other emotional disorders. The dopaminergic innervation from the VTA to PFC strongly affects the PFC’s regulation of cognition and emotion [48].

Acute nicotine administration increased the dopamine level in the PFC of mice with withdrawal in a dose-dependent manner. This is consistent with the findings of Livingstone et al. that nicotine can regulate the release of dopamine in the PFC by activating β2 and α7 nAChRs [49]. The results showed that the dopamine levels in the PFC (*p* < 0.001) and VTA (*p* < 0.01) of the L-NIC+CAF group were significantly higher than that of the L-NIC group. This may be related to the fact that caffeine eliminates the inhibitory effect of adenosine on dopaminergic projection neurons in the VTA and increases dopaminergic input in the frontal cortex [20]. These results could explain the significant decrease in behavioral anxiety and depression caused by withdrawal, suggesting that the combination of caffeine and nicotine returned dopamine levels to normal in mice.

## 5. Conclusions

The results showed that, compared with nicotine alone, the combination of caffeine and nicotine significantly improved the movement ability, anxiety, depression, and pain sensitivity of mice with nicotine withdrawal. Caffeine combined with nicotine also enhanced the memory ability and preference for novel things. The dopamine levels were low in the PFC and VTA of mice with nicotine withdrawal, which may have induced anxiety and depressive behaviors. These dopamine levels increased significantly after treatment with the caffeine and nicotine combination, leading to alleviation of anxiety and depressive behavior. In summary, a combination of caffeine and nicotine effectively relieved nicotine withdrawal syndrome. These results could provide useful insights for further research and clinical practice and could help to establish a more tolerable smooth and effective way to help people quit smoking.

## Data Availability

The original contributions presented in this study are included in the article; further inquiries can be directed to the corresponding author.
